# On the Cause and Curability of Locomotor Ataxia

**Published:** 1884-10

**Authors:** 


### On the Cause and Curability of Locomotor Ataxia.

This subject has been a bone of contention among specialists for some years. First, the cause. It was formerly said that locomotor ataxia was almost never caused by syphilis, but it was due to exposure to wet, severe weather and excessive venery. It is now claimed, and apparently with great foundation, that the cause of locomotor ataxia is certainly specific in about seventy per cent. of all cases. This idea was first advanced by Erb and is confirmed by many noted specialists. Of the few cases seen by the writer, all but one were due to syphilis and this one was traced to exposure to wet, his occupation being that of a sailor. The remaining thirty per cent. of the cases analyzed by Erb were due to exposure and to excessive venery. It is also known that metallic poisoning is a factor in the cause of this disease. But while most of the specialists now agree as to the cause, there still remains a wide difference of opinion as to the curability. Some would say, with Reynolds, that "the prognosis of the disease is, unhappily, full of gloom. Usually, without doubt, the course is slowly but steadily downward." Moreover, it is not impossible to find cases in which the conditions have remained stationary or have changed for the better. "Cases of this kind, it is true, are not very common, but they are to be met with. I myself can testify to the existence of several of them." Others fully agree with Ross that "locomotor ataxia is a serious disease, although the prognosis is not always so serious as it was once thought to be. A certain small percentage end in recovery, and, in many instances, the disease may be arrested for years. The prognosis is favorably influenced by the absence of any hereditary predisposition to the disease or of a neuropathic constitution, by a slow development and moderate intensity of the symptoms, especially of the sensory disturbances, by the patient being in a comfortable condition in life, and by the favorable effect of treatment."

Again, physicians claim a great percentage of recoveries, and maintain that in the early stages it is easily influenced by treatment. Even after the ataxic symptoms have become well developed, the disease can be arrested, and the patient be able to continue his daily occupation. The journals of nervous and mental diseases have contributed, lately, to the literature of this subject, some very interesting and instructive reading. It will be seen that the most of those who study this disease are fully committed to the theory and belief that it is curable. Dr. Hughes, in the last number of the *Alienist and Neurologist*, confirms the theory that this disease is due to syphilis, to exposure and damp. As to its curability, he says: "Recent clinical observations force us to the conclusion, from which we cannot escape, that not only posterior spinal sclerosis, but multiple cerebral sclerosis is either actually curable, or that they both have such symptomatic counterfeits as to render it impossible to distinguish the false from the genuine during life." He reports cases where all the symptoms of locomotor ataxia were present, and, under treatment, had disappeared. The conclusion he reaches is that "these evidences are not alone sufficient to indubitably establish the existence of posterior spinal sclerosis, though they plainly indicate locomotor ataxia as we often see it clinically presented, and point to a condition of the spinal cord, which, if it proceed to dissolution, is found, on post mortem examination, to be one of sclerosis of the posterior columns of the cord."

Dr. Philip Zenner, of Cincinnati, has also lately reported, in the *Cincinnati Lancet and Clinic*, cases of recovery from well-marked symptoms of locomotor ataxia; and Erb, in his work on diseases of the spinal cord, reported cases of locomotor ataxia cured. One of these, in whom the usual symptoms, anæsthesia, ataxia, etc., had been manifested and later disappeared, excepting a slight bladder trouble, after an apparent recovery of about eight years, suddenly died of acute poisoning. An examination of the cord made at this time revealed the degenerative changes, in Burdoch's columns, characteristic of this disease. So, in this case, the symptoms had disappeared, while the pathological changes which produced them had remained. Here we must attribute the improvement, as Dr. Zenner, from whom we extract this reference, does, to vicarious function, certain nerve fibres in the cord assuming the function of those which had been destroyed. "Indeed, clinical observation does establish the curability of the symptom grouping which we have been accustomed to regard as locomotor ataxia, all that is lacking being the cadaveric affirmations of diagnostic accuracy, and this we cannot have in recovered cases." In June last, at the meeting of the American Neurological Association in New York, Dr. G. M. Hammond read a paper: "Can Locomotor Ataxia be Cured?" In this paper he calls attention to the cure of several cases of locomotor ataxia, but without any apparent reason comes to the conclusion: "That these are not cases of true locomotor ataxia."

He says, in conclusion:

"That congestion of the posterior half of the spinal cord may give rise to most, if not, all of the symptoms of locomotor ataxia.

"That it is impossible during life to make a differential diagnosis between posterior spinal sclerosis and posterior spinal congestion.

"That there is no evidence to show that sclerosis, once existing in the spinal cord, has ever been removed.

"That those cases of so-called locomotor ataxia which have been cured are simply cases of spinal congestion more profound in the posterior half of the cord."

The possibility of spinal congestion giving rise to all the symptoms of this most serious disease will need much more conclusive proof than the statement, to be believed by any one, but those who can add to scientific research a very vivid imagination. Many specialists and the author of this review are firm in the belief that there may be such a condition as anæmia and hyperæmia of the cord; but the symptoms would be of a much slighter nature. Many specialists, however, claim the existence of these states, to such a degree as to produce a certain group of symptoms, definite enough to be demonstrated by clinical observation, to be mere fancy. Gowers, in his recent work on the spinal cord, says, on page 73: "It will be observed that I have said nothing of anæmia of the cord or hyperæmia of the cord. In current descriptions of the symptoms of these conditions, I cannot help thinking that a vigorous scientific imagination has contributed much more than observation has supplied. The only practical knowledge of the effects of anæmia and hyperæmia of the cord, is, that they seem capable of causing such disturbances of the sensory structures as reveals itself in subjective sensations of tingling, pins and needles, and the like, and, perhaps, also, some impairment of motor conduction." From this and many more like statements, it would seem that the theory of congestion was not proven and needs further confirmation. Second, how can it be possible to have a congestion of the posterior half of the cord? Third, the statement that sclerosis cannot be removed is certainly true. But cases are on record where the ataxic symptoms have disappeared; either this is due to "certain nerve fibres in the cord assuming the function of those which had been destroyed," or, the portion of cord affected had not entirely gone on to dissolution, and was restored by proper treatment. These explanations seem very much more reasonable than that of congestion.

Dr. Bartholow, of Philadelphia, in discussing this paper, said: "I quite agree with the author of that paper that cases of locomotor ataxia can be cured; but what kind of cases? I was once sharply criticised for reporting a case of locomotor ataxia accompanied by the statement that it could be cured by large doses of iodide of potassium, but it was a case of mercurial ataxia perfectly well developed, which got well. I have no doubt that as the metals are so largely introduced into our common modes of living, there are a great many cases of so-called locomotor ataxia, which are examples of metallic poisoning. I think, also, we should draw a distinction between two classes of cases, undoubtedly syphilitic. Syphilitic gummata of the spinal cord will produce symptoms of locomotor ataxia, and iodide of potassium will remove them. But there is a condition of the system induced by syphilis in its chronic form, favorable to changes in the connective tissue, changes known as sclerosis, in which the results are altogether different, and we should make a distinction, with regard to conclusions drawn with reference to the action of remedies, between those cases due to gummata, and those due to syphilis, which acts, secondarily, as a cause. In that restricted sense I believe locomotor ataxia can be cured. Furthermore, I believe that by the use of remedies, unquestionable cases of locomotor ataxia can be arrested. There is, however, a great difference between arrest and restoration to *status in quo*."

Dr. Mills, of Philadelphia, said: "I have not seen cases of what might be termed the regular form of the disease cured.

When I say regular form, I mean that type which we recognize pathologically as posterior spinal sclerosis. I have seen, however, several cases with ataxic symptoms relieved and cured. I believe those cases with ataxic symptoms, in which there is a distinct syphilitic history, will be relieved by the energetic use of anti-syphilitic remedies." From these facts, we are forced to the conclusions:

First, that the theory of hyperæmia of the cord producing symptoms which cannot be differentiated from true locomotor ataxia, is unproven and improbable.

Second, that the condition of the spinal cord producing ataxic symptoms is frequently relieved and cured.

Third, that many cases of locomotor ataxia, when the disease has progressed to a marked degree, have been arrested for years.

Fourth, that locomotor ataxia, due to syphilis, which forms a vast number of cases, is readily relieved and cured.

These considerations should make us ever careful in the diagnosis and treatment of this disease, once considered hopeless, now hopeful, and when our therapeutic power is equal to our knowledge of the symptoms of this disease, "we shall have added another therapeutic triumph to the many marvelous successes of the past." F. S. C.

